# A radiologic review of hoarse voice from anatomic and neurologic perspectives

**DOI:** 10.1186/s13244-019-0786-7

**Published:** 2019-11-18

**Authors:** Simone Montoya, Anthony Portanova, Alok A. Bhatt

**Affiliations:** 1 University of Rochester Medical Center, Rochester, NY 14642 USA; 2 Mayo Clinic, Jacksonville, FL 32224 USA

**Keywords:** Hoarseness, Vocal cord dysfunction, Recurrent laryngeal nerve, Larynx

## Abstract

The differential diagnosis for hoarseness is extensive and includes a multitude of etiologies that span a large geographic area from the brainstem to the mediastinum. Therefore, localizing a causative lesion can be extremely difficult for clinicians and radiologists alike. In this review, we will first discuss the normal anatomy of the larynx and its innervation via the vagus and recurrent laryngeal nerves. We will then proceed with a guided tour of the various infectious/inflammatory, neoplastic, congenital, and traumatic/iatrogenic causes of hoarseness subdivided by anatomic location (brainstem, skull base, carotid sheath, thyroid, larynx, and superior mediastinum). Along the way, we will discuss the various cross-sectional imaging modalities best suited to detect the often subtle signs of recurrent laryngeal nerve injury. With thorough knowledge of these entities, radiologists can impact patient care by suggesting the appropriate imaging test and tailoring their search patterns to detect the subtle findings of laryngeal dysfunction.

## Key points

- The clinical symptom of hoarseness carries with it an extensive differential diagnosis, which is not confined to neoplastic etiologies.
- The anatomic course of the recurrent laryngeal nerves differs bilaterally, which impacts the geographic extent of imaging coverage required to diagnose causative lesions.
- Laryngeal dysfunction can be caused by lesions located anywhere from the brainstem to the mediastinum.

## Introduction

Dysphonia affects one-third of people at some point in their lifetime, and an estimated one in 13 adults experiences hoarse voice annually [1]. Hoarse voice is often used interchangeably with dysphonia; however, the former is a symptom while the latter is a clinical diagnosis. Dysphonia is a broad term for impaired vocal production, including altered quality, pitch, loudness, or effort. Causes for dysphonia can be divided into organic and functional etiologies. Organic dysphonia is due to a physiologic change in vocal production, and is further divided into structural and neurogenic dysphonia. Structural etiologies impact a physical change upon the mechanism of vocal production (i.e., the larynx), while neurogenic etiologies effect via the nervous system (i.e., vagus or recurrent laryngeal nerve or their nuclei). Functional dysphonia is due to vocal overuse or abuse, and can have overlap with organic dysphonia.

Any hoarseness not readily attributable to a benign cause (such as an acute upper respiratory infection) and lasting more than 4 weeks should be further evaluated; this is especially important when there are additional coexisting symptoms such as dysphagia, odynophagia, cough, hemoptysis, unilateral ear/throat pain, neck mass, weight loss, or if there are significant risk factors for head and neck cancer [1]. The first line of investigation is laryngoscopy; however, diagnostic imaging comes into play if a cause is not identified or if further evaluation is warranted.

The biomechanics of phonation is a complex process which can be altered by a wide range of local and systemic processes, many which are not readily apparent on laryngoscopy. The head and neck radiologist must be familiar with the anatomy and pathology of the larynx, as well as the complex supporting laryngeal network. Here, we will briefly review the normal anatomy of the structures of phonation, as well as the course of the vagus and recurrent laryngeal nerves, then present important etiologies of dysphonia with case examples by location.

### Anatomy of the larynx

**Fig. 1:**
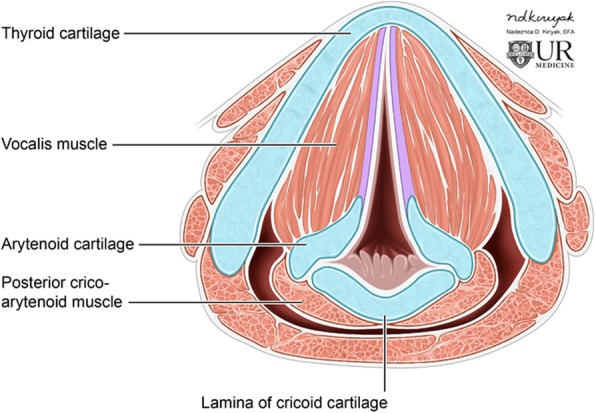
Representative illustration of the larynx at the level of the true vocal folds demonstrating the normal configuration of the cartilaginous structures and intrinsic musculature

The larynx, also known colloquially as the voice box, resides between the anatomic oropharynx and trachea and is subdivided into the supraglottis, glottis, and subglottis. It is comprised of six cartilaginous structures, three which are single—thyroid, cricoid, and epiglottis—and three which are paired—arytenoid, cornicuate, and cuneiform. The glottis contains the true vocal cords, which are double-layered membranous folds which attach to the arytenoid cartilages on either side. The vocal cords are responsible for vocal production via vibration as exhaled air passes through the glottis. The intrinsic musculature is responsible for vocal cord and epiglottic movement, while the extrinsic group provides support to the larynx. The primary intrinsic muscles responsible for vocal cord movement are the cricothyroid, posterior cricoarytenoid, lateral cricoarytenoid, transverse arytenoid, thyroarytenoid, and vocalis (Fig. 1). Aside from the cricothyroid, which is innervated by the superior laryngeal nerve, the muscles of the vocal cords are innervated by the recurrent laryngeal nerve; both nerves are branches of the vagus nerve.

### Innervation of the larynx and vocal cord paralysis

**Fig. 2:**
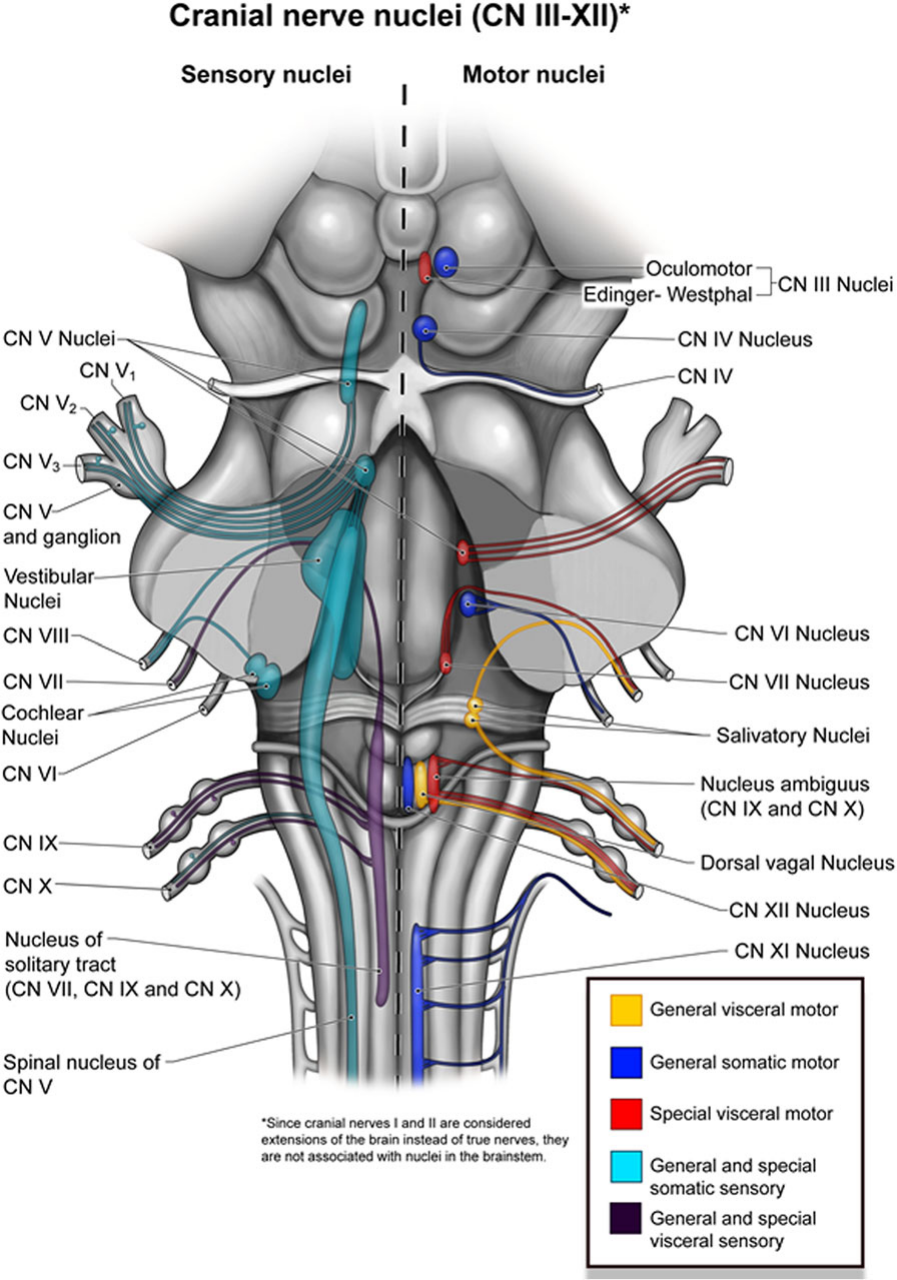
Representative illustration of the brainstem demonstrating the normal configuration of the cranial nerves and their associated nuclei. The vagus nerve originates from the nucleus ambiguus of the medulla

The vagus nerve (CN X) originates from the nucleus ambiguous, located in the medulla oblongata between the medullary pyramid and the inferior cerebellar peduncle (Fig. 2). Upon leaving the medulla, the vagus nerve courses through the lateral cerebellomedullary cistern and exits the skull base via the pars vascularis of the jugular foramen. It then descends the neck within the carotid sheath, coursing between and posterior to the carotid artery (lateral) and internal jugular vein (medial), and enters the superior mediastinum near the aortic arch and origin of the great vessels.

**Fig. 3:**
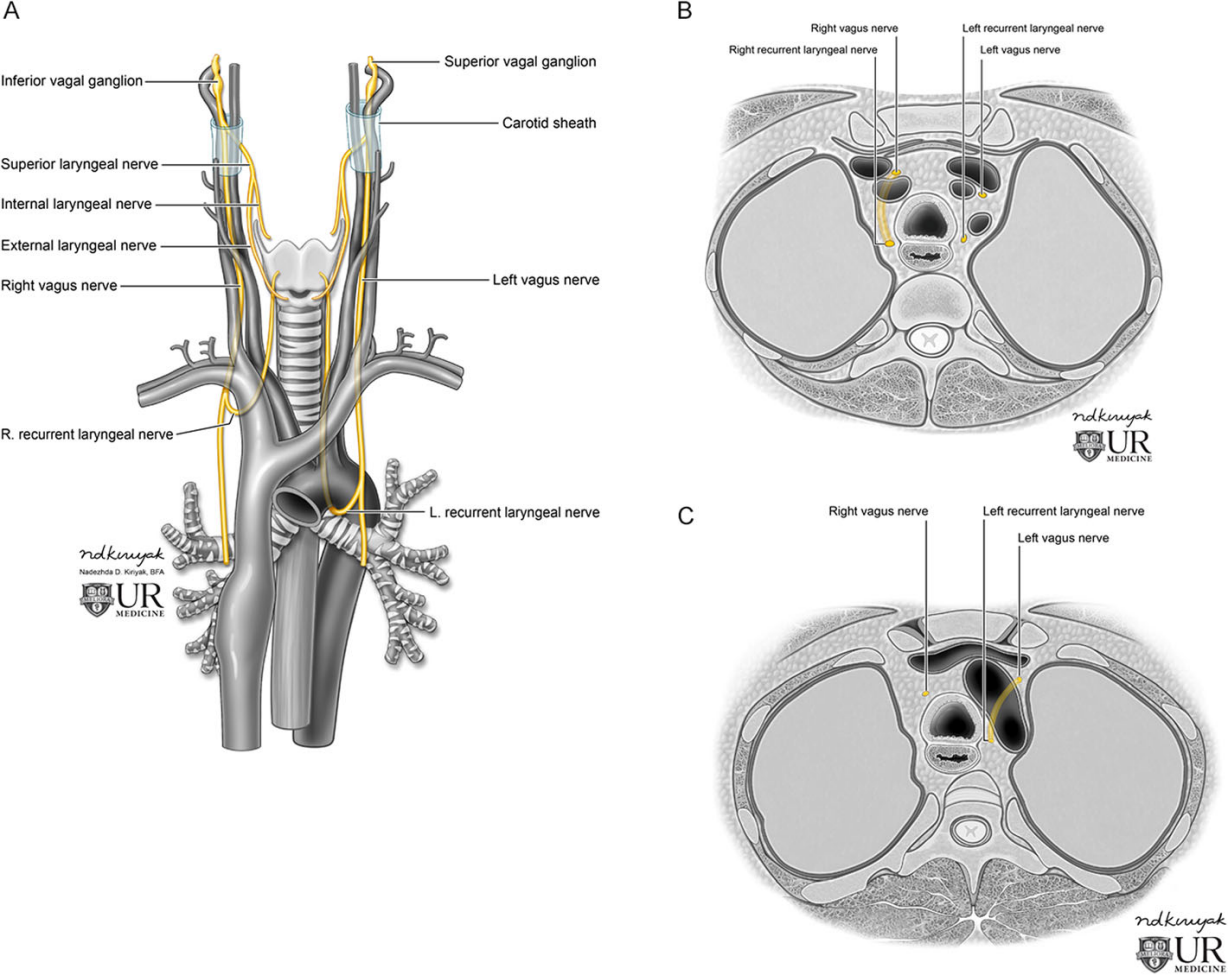
Representative coronal (**a**) and axial (**b**, **c**) illustrations demonstrating the normal course of the bilateral vagus and recurrent laryngeal nerves. The right recurrent laryngeal nerve loops posteromedially under the right subclavian artery (**a**, **b**), and the left recurrent laryngeal nerve loops posteromedially beneath the aortic arch (**a**, **c**)

The recurrent laryngeal nerve (RLN) branches anteriorly off the vagus nerve in the mediastinum. Due to the asymmetry of the aortic arch, the course on either side is slightly different; the right RLN loops posteromedially under the right subclavian artery, while the left RLN loops posteromedially under the arch itself, through the aortopulmonary window. Both then ascend along the tracheoesophageal groove toward the larynx (Fig. 3).

Vocal cord paralysis (VCP) occurs when there is impaired function of either CN X or the RLN. This can occur secondary to multiple disorders which can be characterized by etiology (Table 1) and location (Table 2). Most cases are unilateral and due to a compressive mass, although up to one-third may be bilateral [2]—usually seen in iatrogenic/traumatic injury, but also seen with neurogenic disorders such as amyotrophic lateral sclerosis or closed head injury which can have multifocal lesions. Up to 40% of people with unilateral VCP are asymptomatic at the time of diagnosis. VCP may be accompanied by co-symptomatology of other lower cranial neuropathies depending on the location of the lesion—a mass located from the brainstem to the jugular foramen may present with VCP along with dysphagia due to loss of pharyngeal sensation (CN IX dysfunction) and/or denervation of the trapezius and sternocleidomastoid muscles (CN XI dysfunction), while a mass within the carotid sheath may present with the aforementioned, as well as tongue atrophy and deviation (CN XII dysfunction).

**Table 1:** Differential diagnosis of vocal cord dysfunction by location

Brainstem	Skull base	Carotid sheath	Thyroid	Superior mediastinum	Larynx
Tumor	Meningioma	Glomus vagale	Carcinoma	Tumor/lymphadenopathy	Laryngocele
Infarction	Metastasis	Schwannoma	Iatrogenic	Vascular etiologies	Polyps, cysts
	Glomus jugulare	Trauma	Goiter		Squamous cell carcinoma
					Papillomatosis
					Amyloidosis

**Table 2:** Differential diagnosis of vocal cord dysfunction by etiology

Infection/inflammation	Intrinsic vocal fold lesions	Vocal cord paralysis	Miscellaneous
Acute/chronic laryngitis	Laryngeal polyps/nodules/cysts	Post-tracheal intubation	Laryngocele
Laryngeal amyloidosis	Laryngeal papilloma	Compression of CN X or RLN—mediastinal causes	Laryngeal web
Thyroid disease	Laryngeal cancer	Compression of CN X or RLN—thyroid mass	
Rheumatoid arthritis		Compression of CN X or RLN—skull base	
		Traumatic Injury to CN X or RLN	
		Neurodegenerative disorders (i.e., ALS, Parkinsons)	

## Lesions and disorders of the larynx

### Laryngocele

The laryngeal ventricles of Morgagni are paired structures of the larynx residing between the vocal and vestibular folds; the saccule is an appendageal diverticulum extending vertically from the ventricle between the vestibular fold and the thyroid cartilage, and is responsible for producing mucus that lubricates the vocal cords. Cystic dilatation of the saccule is termed a laryngocele, which may be developmental due to failure of regression after birth, and can also be seen in persons who experience high throat pressures, such as wind musicians, glass blowers, and those with excessive coughing [3]. These are often asymptomatic and found incidentally, but occasionally can present with hoarseness and stridor. Symptoms may be episodic due to intermittent filling and resultant one-way valve effect, or due to infection [4].

**Fig. 4:**
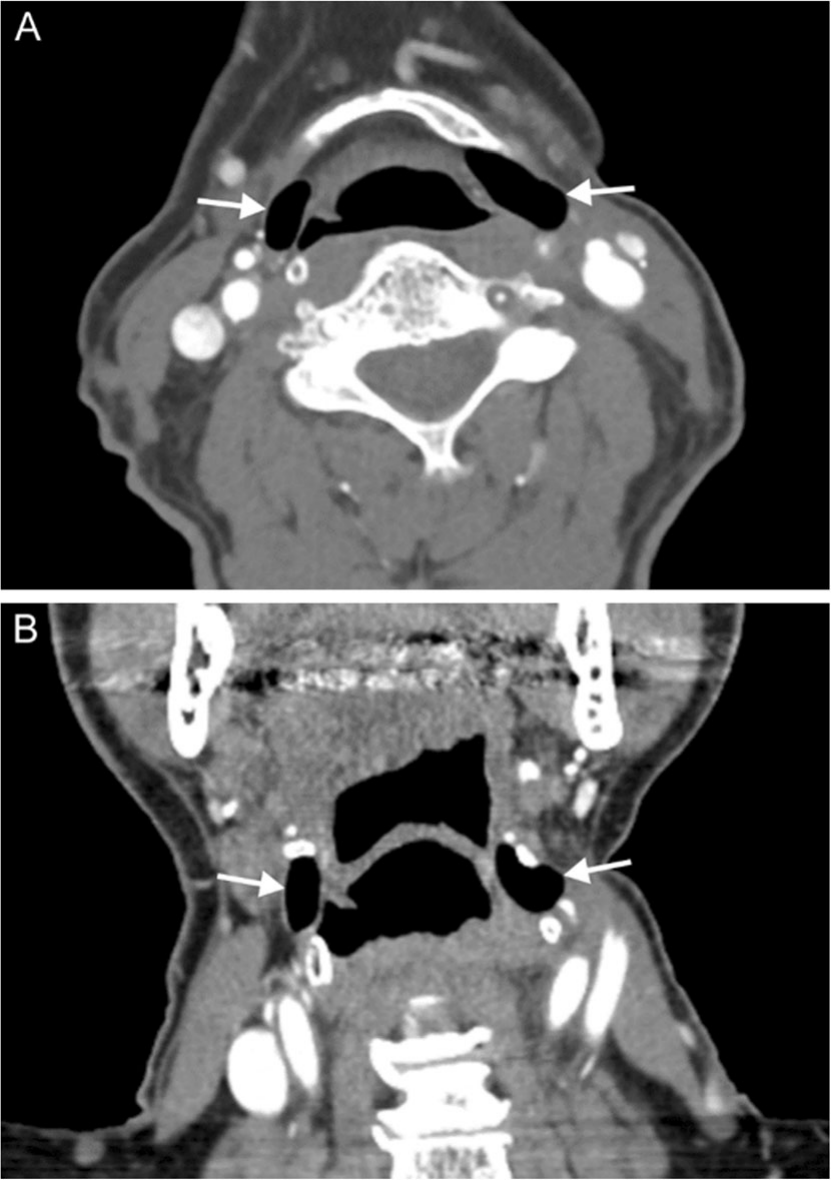
Laryngocele. A 67-year-old man with history of prior tongue base and oropharyngeal squamous cell carcinoma status post radiation completed 8 years prior presents with dysphagia and hoarse voice. Axial (**a**) and coronal (**b**) contrast-enhanced CT images demonstrate bilateral supraglottic laryngoceles (arrows) with mild extralaryngeal extension on the left

On imaging, a laryngocele appears as a thin-walled air- or fluid-filled space lateral to and communicating with the laryngeal lumen; the wall will demonstrate absent or minimal enhancement [5] (Fig. 4). Types of laryngoceles are defined by their relationship to the thyrohyoid membrane—internal and external/mixed [4]. Internal (or simple) laryngoceles are confined to the paraglottic space of the supraglottis, and are found lateral to the thyrohyoid membrane. If the laryngocele extends superiorly through the thyrohyoid membrane into the submandibular space, then it is termed a mixed laryngocele. An external laryngocele does not have an internal component and is rare; it may present as a lateral neck mass and can be intimately associated with the superior laryngeal nerve.

### Benign inflammatory laryngeal lesions

**Fig. 5:**
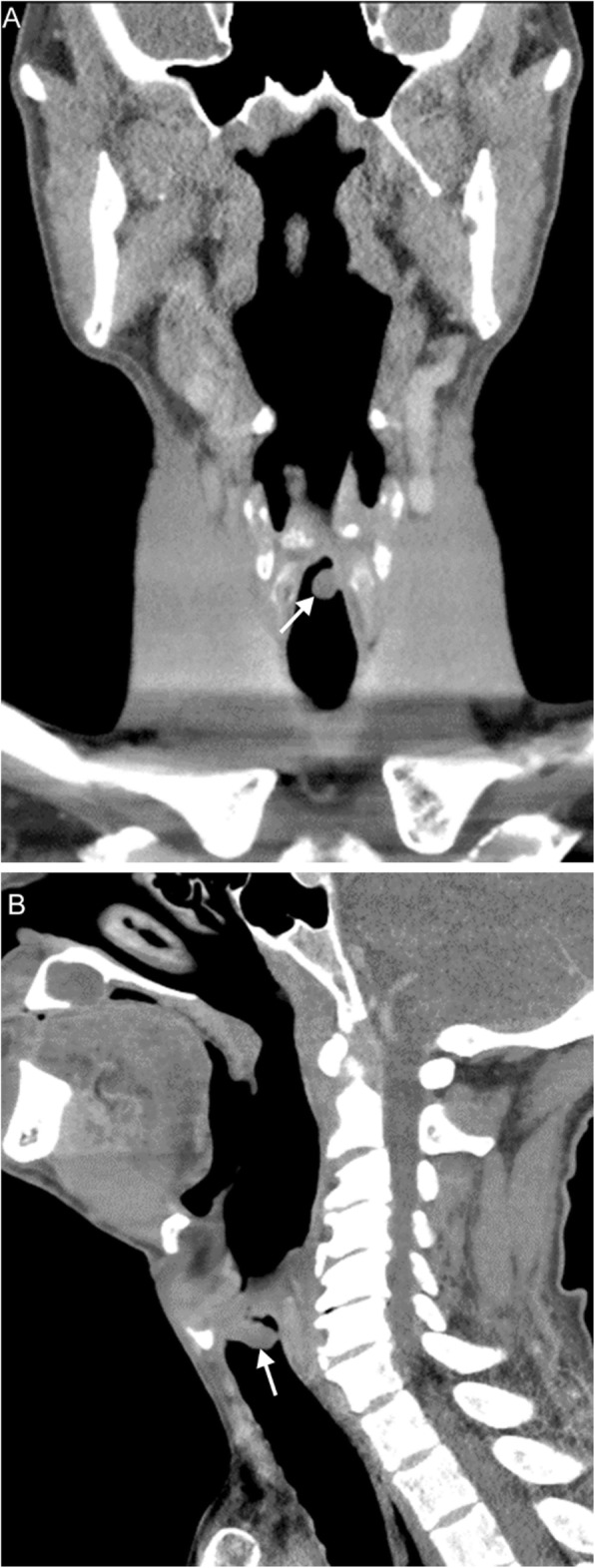
Laryngeal polyp. A 57-year-old man heavy smoker reporting sore throat, hoarseness, and stridor. Coronal (**a**) and sagittal (**b**) contrast-enhanced CT images reveal a polypoid lesion arising from the left wall of the larynx (arrows). Laryngoscopy (not shown) revealed a round mass with ball-valve obstruction of the glottis. Subsequent biopsy confirmed a laryngeal polyp

Repetitive insult to the laryngeal mucosa—such as with singing, yelling, smoking, gastroesophageal reflux—can lead to a number of benign inflammatory lesions, especially of the vocal cords. Although related in etiology, these lesions differ in mechanics of origin and appearance [6, 7]. Vocal cord nodules occur bilaterally and symmetrically at the midpoint of the cords due to high shear force during apposition and subsequent remodeling, similar to a callous. A laryngeal polyp forms after trauma to the submucosal capillary bed, with subsequent exudative inflammatory changes and outpouching of the mucosa (Fig. 5). A laryngeal cyst can appear similar to a laryngocele and may be of two types: retention, due to glandular obstruction and resultant trapped secretions, or epidermoid, which are congenital or due to trauma. Reinke’s edema is not a discrete lesion, but is diffuse edema of the vocal cords due to fluid accumulation between the membranous folds (Reinke’s space). These lesions do not warrant imaging as they are readily seen by laryngoscopy; however, they may be seen incidentally on head and neck imaging obtained for other reasons [8].

### Squamous cell carcinoma

Squamous cell carcinoma (SCC) is by far the most common (98%) primary tumor of the larynx, and can also secondarily involve the larynx when the primary is elsewhere in the oropharynx. SCC generally occurs in men over 50 and was previously associated with smoking and alcohol abuse; however, SCC related to human papilloma virus (HPV) is now more prevalent, and is seen in the younger population with better prognosis. Classification is based on subsite of location in relation to the glottis—supraglottic (20–30%), glottic (50–60%), subglottic (5%), and transglottic (spanning two or more subsites)—and presentation depends on the subsite involved. Glottic lesions often present earlier with dysphonia or aspiration, while subglottic lesions typically present with dyspnea and/or stridor. Purely supraglottic lesions are asymptomatic, thus usually present later with symptoms due to lymphadenopathy or trans-spatial spread, such as tender neck mass, sore throat, dysphagia/odynophagia, or referred ear pain.

**Fig. 6:**
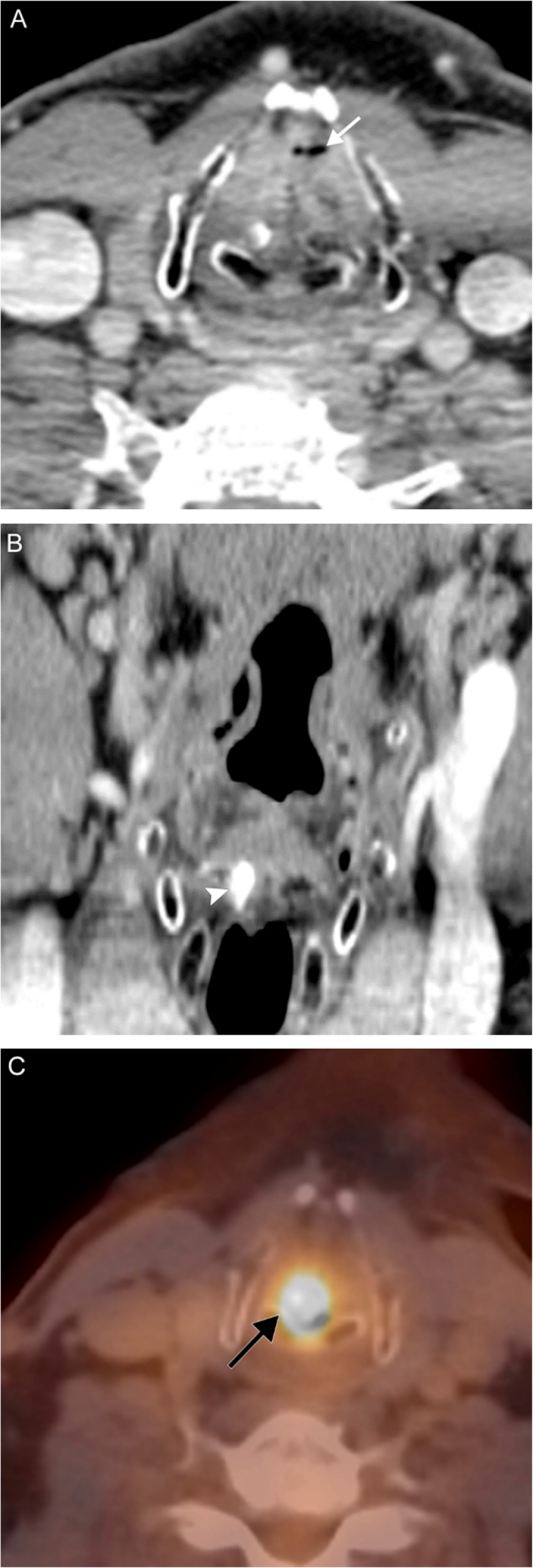
Laryngeal carcinoma. A 59-year-old man with 45-pack-year smoking history presenting with 6 weeks of hoarseness. Axial (**a**) and coronal (**b**) contrast-enhanced CT images reveal near-complete obliteration of the laryngeal airway by a polypoid soft tissue mass, which extends inferiorly into the subglottic airway. Note the trace residual airway remnant anteriorly (white arrow). There is asymmetric high attenuation of the right arytenoid cartilage (white arrowhead), consistent with sclerosis and involvement of the lesion. Axial fusion PET/CT image (**c**) reveals a hypermetabolic mass centered at the larynx (black arrow). Biopsy revealed squamous cell carcinoma

**Fig. 7:**
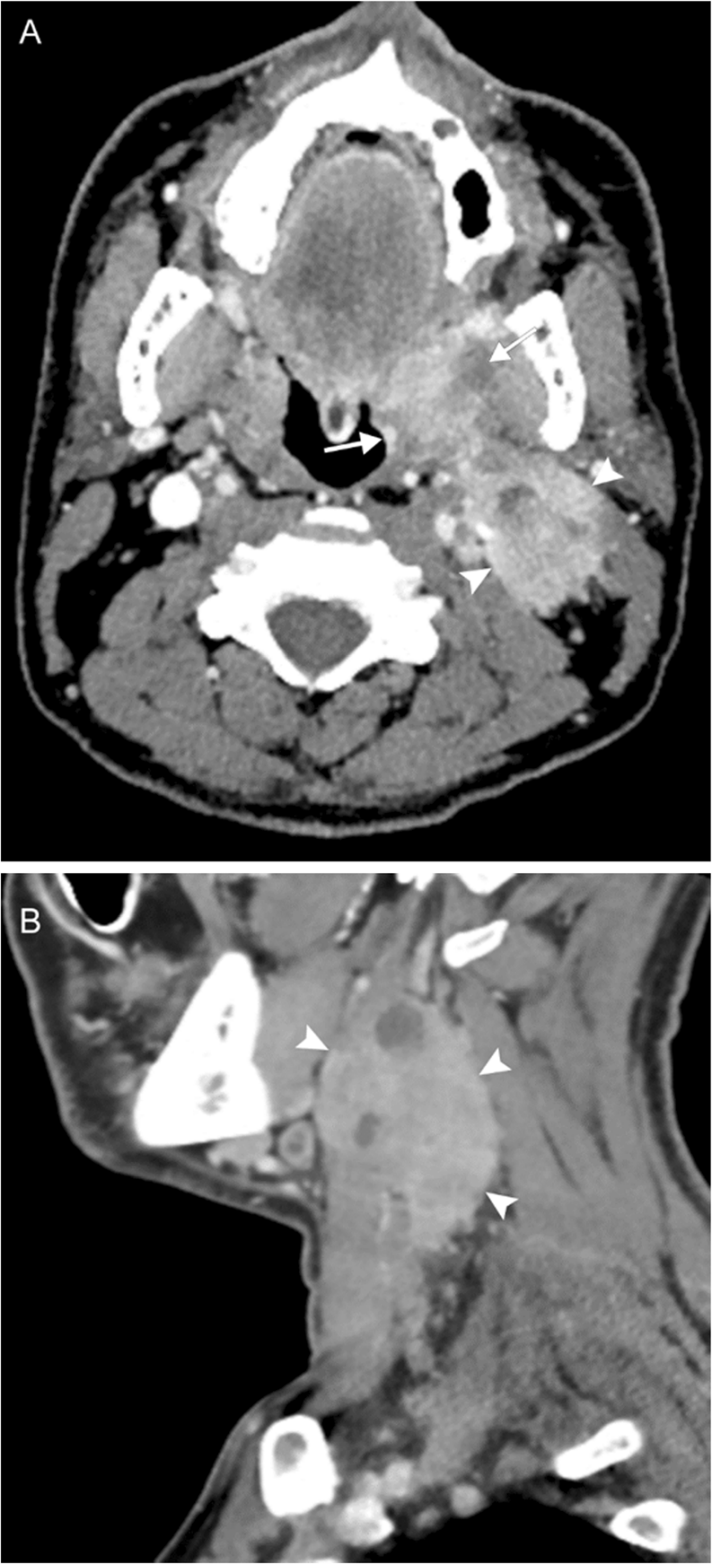
Oropharyngeal squamous cell carcinoma. A 40-year-old man presenting with tender neck mass, dysphagia to solid food, odynophagia, and voice changes. Axial (**a**) and sagittal (**b**) contrast-enhanced CT images reveal an enhancing mass at the left oropharynx (arrows) and extensive left-sided lymphadenopathy, including a large level II nodal conglomerate with central areas of necrosis (arrowheads). Biopsy revealed this to be human papilloma virus (HPV)-mediated squamous cell carcinoma. Voice change was attributed to encroachment upon the carotid space resulting in vagal nerve irritation.

As laryngoscopy can be performed by the otolaryngologist, imaging is used for staging rather than diagnosis. SCC appears as an enhancing soft tissue mass on both computed tomography (CT) and MRI; either may be used for disease surveillance. The lesion is usually isoattenuating to the surrounding mucosa on CT, but may be identified due to architectural distortion and loss of tissue planes (Figs. 6 and 7). On MRI, the lesion is usually T1-hyperintense, T2-hyperintense, and demonstrates high STIR signal due to edema. Positron emission tomography (PET) may be used in conjunction to identify metastatic lymphadenopathy or post-treatment occurrence.

Small lesions may be treated locally with laser or radiation ablation; larger tumors usually necessitate laryngectomy and radiation therapy, although supraglottic tumors without vocal cord fixation may be treated with voice-sparing surgery. Compared to similarly staged SCC elsewhere in the head and neck, laryngeal SCC portends a better prognosis, but 15–20% will develop a second primary site SCC that is most certainly fatal.

### Recurrent respiratory papillomatosis

**Fig. 8:**
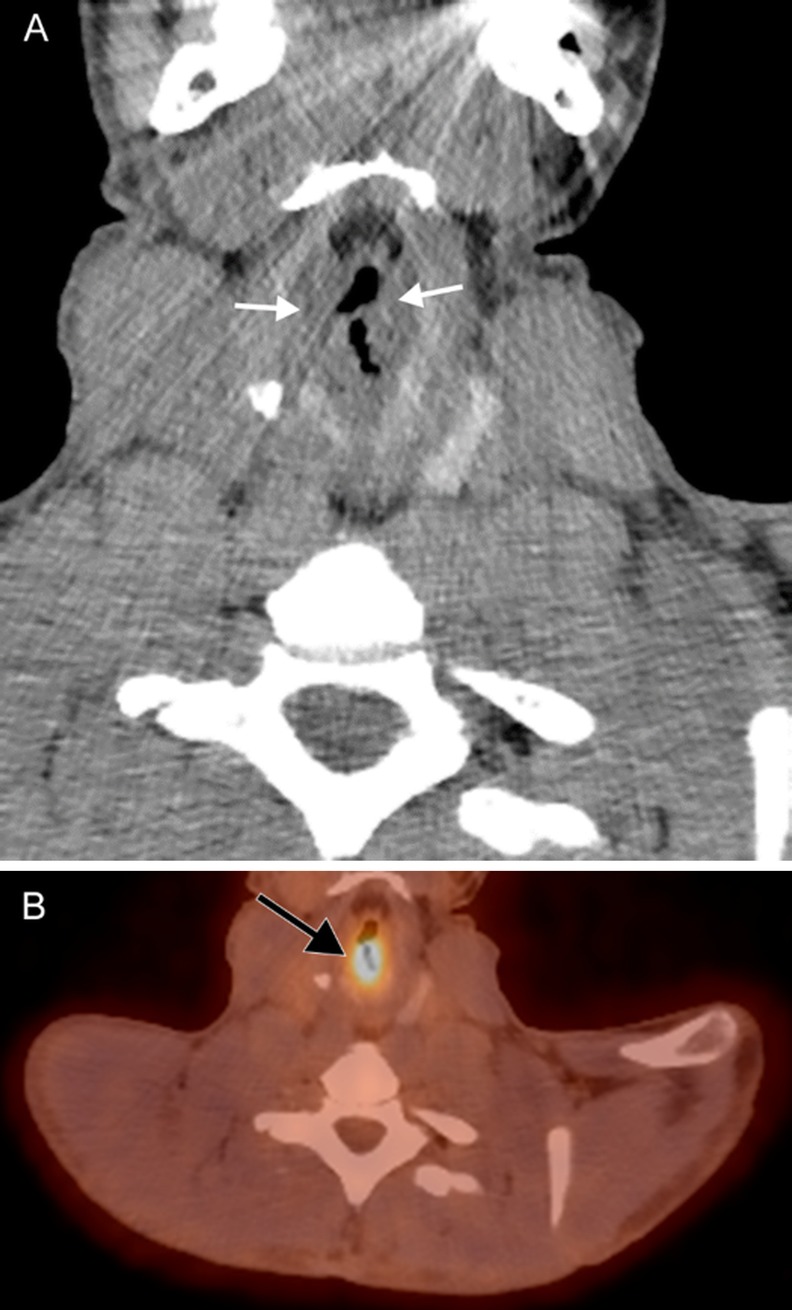
Laryngeal papillomatosis. A 26-year-old man with recurrent tracheal/laryngeal papillomatosis status post tracheal diversion and radiation therapy. A coronal contrast-enhanced CT image (**a**) reveals nodular soft tissue attenuation (white arrows) replacing the laryngeal structures. Subsequent axial attenuation-corrected PET image (**b**) reveals marked FDG-avidity (black arrow)

Respiratory papillomatosis is caused by human papilloma virus (HPV); it has a propensity for the larynx but can also occur elsewhere in the airway [9]. It is the most common laryngeal tumor in children, but can be seen in adults. In juvenile-onset respiratory papillomatosis, HPV is transmitted from a mother to her child during passage through the birth canal; an infected mother has a 1% risk for transmission to her child. In adult-onset respiratory papillomatosis, transmission is thought to occur via oral sex [10, 11]. The most common strains in respiratory papillomatosis are HPV 6 and HPV 11; however, HPV 16 carries the highest risk for malignant transformation. Respiratory papillomatosis results in airway obstruction, which may be due to either blockage by an intraluminal lesion or infiltration of the structural tissues; tissue infiltration can also result in dysphonia due to morphologic alteration of the laryngeal components. Imaging characteristics are nonspecific; intraluminal lesions tend to be sessile and verrucoid, while infiltrative lesions appear as irregular soft tissue thickening. Lesions tend not to enhance avidly or at all; however, they will demonstrate fluorodeoxyglucose (FDG)-avidity on PET (Fig. 8). Treatment is antiretroviral therapy and surgical resection, possibly complemented by airway reconstruction in cases of wide resection necessitated by large lesions or extensive infiltration; as implied by the name, however, papillomatosis tends to recur [12].

### Laryngeal amyloidosis

**Fig. 9:**
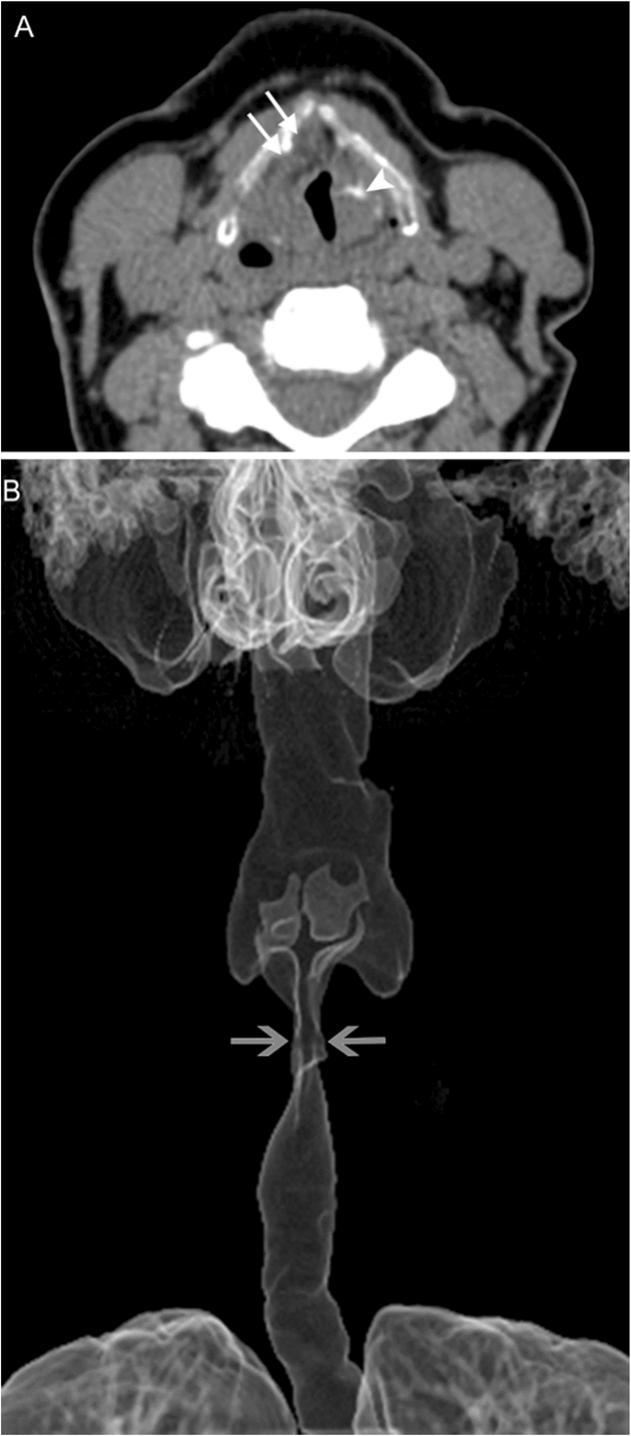
Laryngeal amyloidosis. A 69-year-old woman with recurrent laryngeal amyloidosis despite multiple interventions. Axial CT image (**a**) reveals marked soft tissue thickening of the laryngeal structures extending into the right pre/paraglottic space (white arrows) representing amyloid infiltration, and dystrophic calcification (white arrowhead) representing post-treatment change. The true vocal cords are not identifiable. 3D surface volume rendering (**b**) demonstrates marked narrowing of the glottic airway (gray arrows)

Amyloidosis is the extracellular deposition of a variety of fibrils that are composed of various low molecular weight subunits of proteins that are found normally in blood serum; this abnormal tissue is called amyloid. Laryngeal amyloidosis is a rare entity, accounting for only 1% of laryngeal masses [13, 14]. Amyloidosis is a slow progressive process that results in hoarseness due to tissue infiltration by amyloid, which alters the structure and mechanics of the vocal apparatus. Deposition in laryngeal tissue is most often a localized process, but systemic amyloidosis with laryngeal involvement is usually due to a monoclonal plasma cell dyscrasia. Imaging findings in amyloidosis are nonspecific and result from architectural distortion of laryngeal structures due to tissue infiltration (Fig. 9). Treatment consists of endoluminal microsurgical resection or debulking, and often requires multiple sessions due to extent and recurrence [15].

## Brainstem lesions

### Hypertrophic olivary degeneration

The dentato-rubro-olivary tract, also known as the myoclonic or Guillain-Mollaret triangle, connects the inferior olivary nucleus (ION) in the medulla to the ipsilateral red nucleus in the midbrain and contralateral dentate nucleus in the cerebellum via the central tegmental, olivocerebellar/olivodentate, and dentorubral tracts. A lesion disrupting any of these pathways can result in deafferentation of the inferior olivary nucleus, either contralateral (lesion of the superior or inferior cerebellar peduncle) or ipsilateral (lesion of the central tegmentum). Due to trans-synaptic degeneration, there is cytoplasmic vacuolar degeneration, along with glial hypertrophy and astrocytic proliferation, initially resulting in enlargement of the ION; this hypertrophy is unique to these lesions, although the olive eventually becomes atrophic. Often the offending lesion is hemorrhagic, but may be ischemic or demyelinating [16].

The classic clinical finding in hypertrophic olivary degeneration (HOD) is palatal myoclonus, which is involuntary rhythmic movement involving the oropharynx, typically the levator palatini but may also include the larynx; ocular myoclonus and tremor or ataxia of the upper extremity may also be present. Symptoms usually present several months after the primary insult, but the patient may also be asymptomatic.

**Fig. 10:**
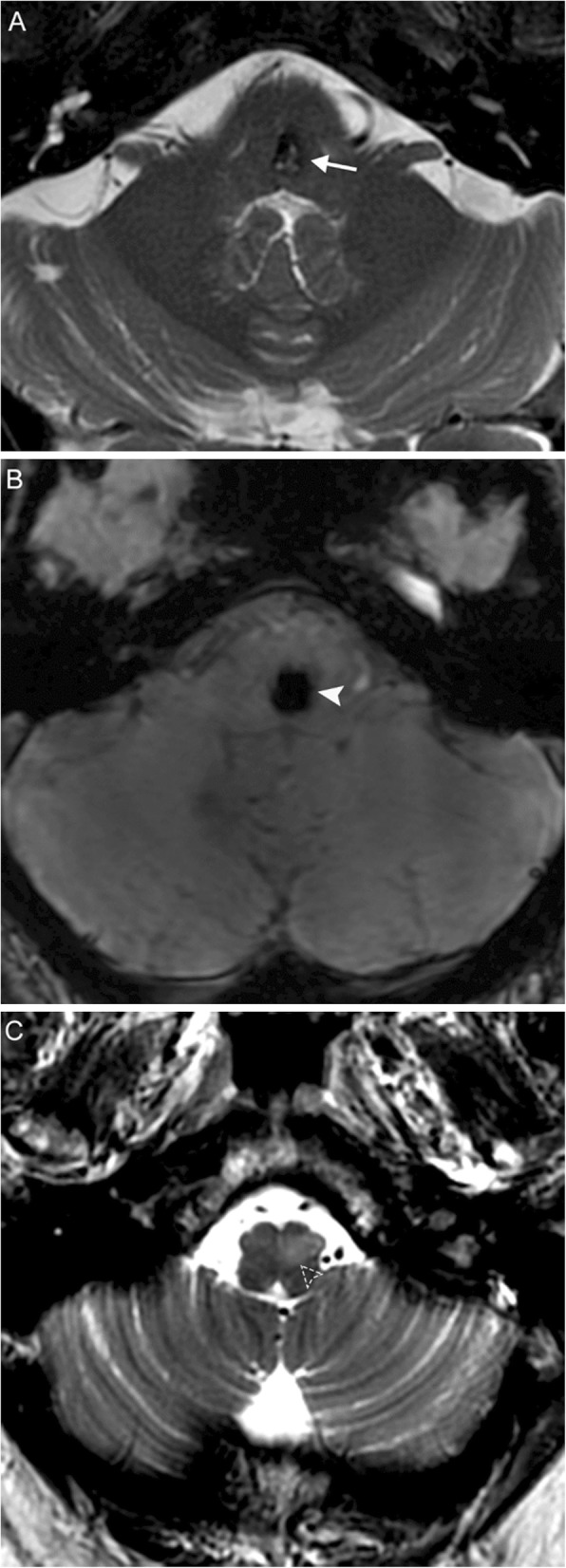
Hypertrophic olivary degeneration. A 69-year-old man with history of hypertension presenting with new onset palatal myoclonus, hoarse voice, and ataxia. Axial T2 (**a**) and susceptibility-weighted (**b**) MR images reveal a lesion at the left parasagittal pontomedullary junction at the level of the facial colliculus demonstrating bubbly high T2 signal internally surrounded by a rim of low T2 signal intensity (arrow) and associated blooming (solid arrowhead), compatible with a cavernous malformation. Thin section axial T2 image (**c**) reveals hyperintensity and expansion of the left inferior olivary nucleus (dashed arrowhead), consistent with hypertrophic olivary degeneration. Lesions involving the dento-rubro-olivary tract (such as this cavernous malformation) can lead to hypertrophic olivary degeneration

MRI is characterized by distinct phases of progression. The ION initially appears normal, then develops T2-hyperintensity after 1 month. After several months to a few years, the ION remains T2-hyperintense and also becomes hypertrophic; this hypertrophy eventually resolves, and the ION becomes atrophic. Contrast enhancement is atypical (Fig. 10).

### Lateral medullary syndrome

**Fig. 11:**
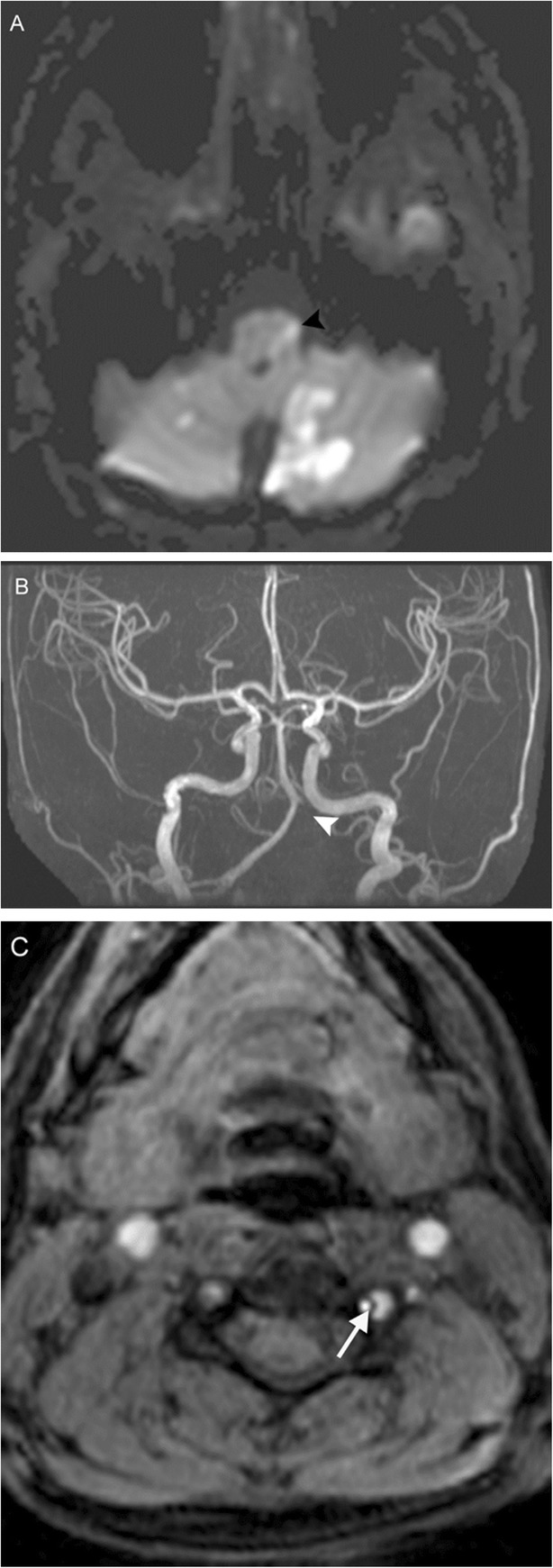
Lateral medullary infarction. A 33-year-old woman with history of migraines presenting with acute onset vertigo, nausea, weak voice/swallow, and left extremity sensorimotor deficits after chiropractic manipulation. Diffusion-weighted MRI (**a**) reveals numerous acute embolic infarctions involving the bilateral cerebellar hemispheres and bilateral thalami, with notable involvement of the left PICA territory including the left lateral medulla (black arrowhead). TOF MRA MIP image (**b**) reveals absence of flow related enhancement within the left V4 segment and PICA (white arrowhead). Axial TOF image (**c**) reveals small caliber flow within the residual true lumen (white arrow), with surrounding crescentic lower signal in the false lumen. This constellation of findings is consistent with left vertebral artery dissection with showering of emboli resulting in infarction

The posterior inferior cerebellar artery (PICA) territory includes the inferior occipital surface of the cerebellar hemisphere and inferior vermis, while perforators off the intracranial vertebral (V4 segment) and basilar arteries supply the brainstem. Occlusion of the distal V4 segment or PICA can result in infarction of the lateral medulla (Fig. 11); this is characterized by a constellation of symptoms known as lateral medullary or Wallenberg syndrome [17]. Due to involvement of the nucleus ambiguus, patients will have ipsilateral bulbar weakness affecting the muscles of the soft palate and pharynx, resulting in dysphonia, dysphagia, and dysarthria. Other symptoms include autonomic dysfunction, ipsilateral Horner’s sign (meiosis, ptosis, enophthalmos, anhydrosis), ipsilateral facial and contralateral truncal/appendicular sensory disturbance, and vestibulocerebellar symptoms such as ataxia and vertigo (patients will “fall toward the lesion”).

## Lesions at the jugular foramen

The jugular foramen is situated at the junction between the petrous temporal and occipital bones and is divided into two compartments. The pars nervosa is smaller and anteromedial, and contains the inferior petrosal sinus and CN IX. The pars vascularis is larger and posterolateral, and contains the jugular bulb and CNs X and XI.

**Fig. 12:**
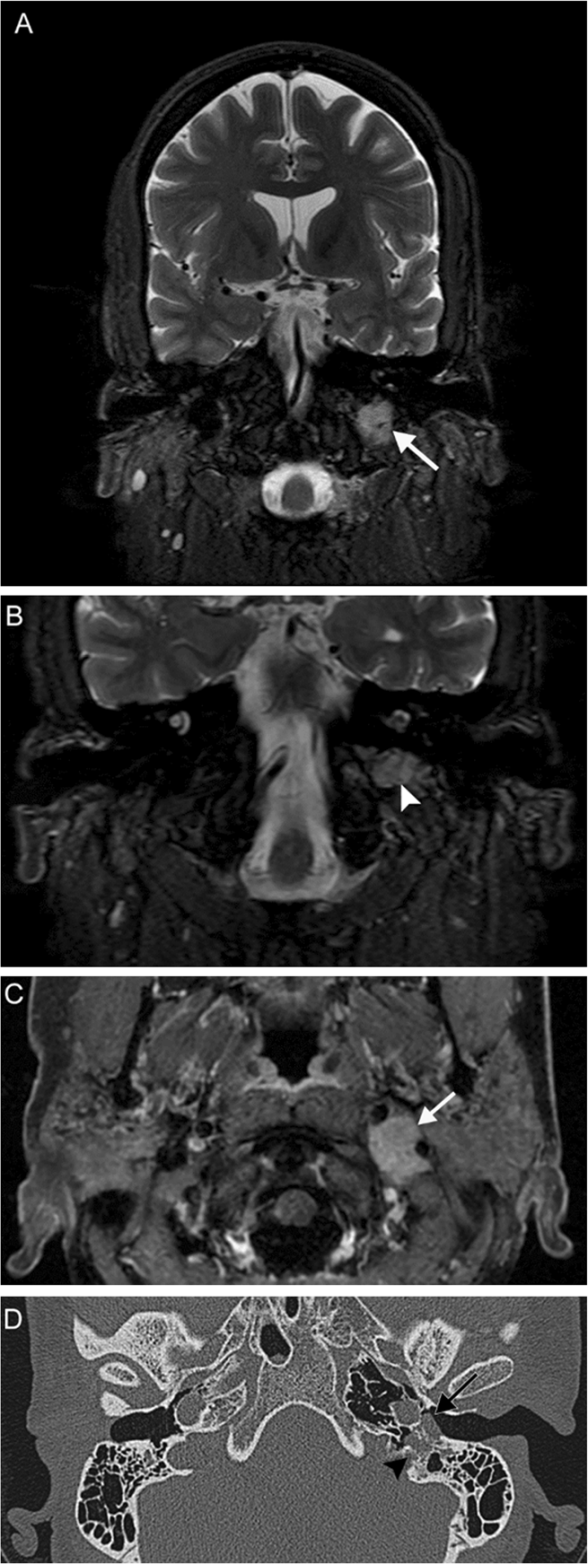
Jugulotympanicum paraganglioma. A 49-year-old woman with voice change and pulsatile tinnitus. Coronal T2-weighted MR images (**a**, **b**) and axial contrast-enhanced T1 image (**c**) demonstrate a T2-hyperintense, enhancing mass centered at the left jugular bulb (white arrows) extending into the hypotympanum (white arrowhead). Axial CT of the temporal bones (**d**) reveals abnormal soft tissue in the left middle ear (black arrow) with osseous 'moth-eaten' destruction at the jugular foramen (black arrowhead); the cochlear promontory in preserved (not shown). Based on location and appearance, findings are consistent with a jugulotympanicum paraganglioma (glomus jugulotympanicum tumor)

**Fig. 13:**
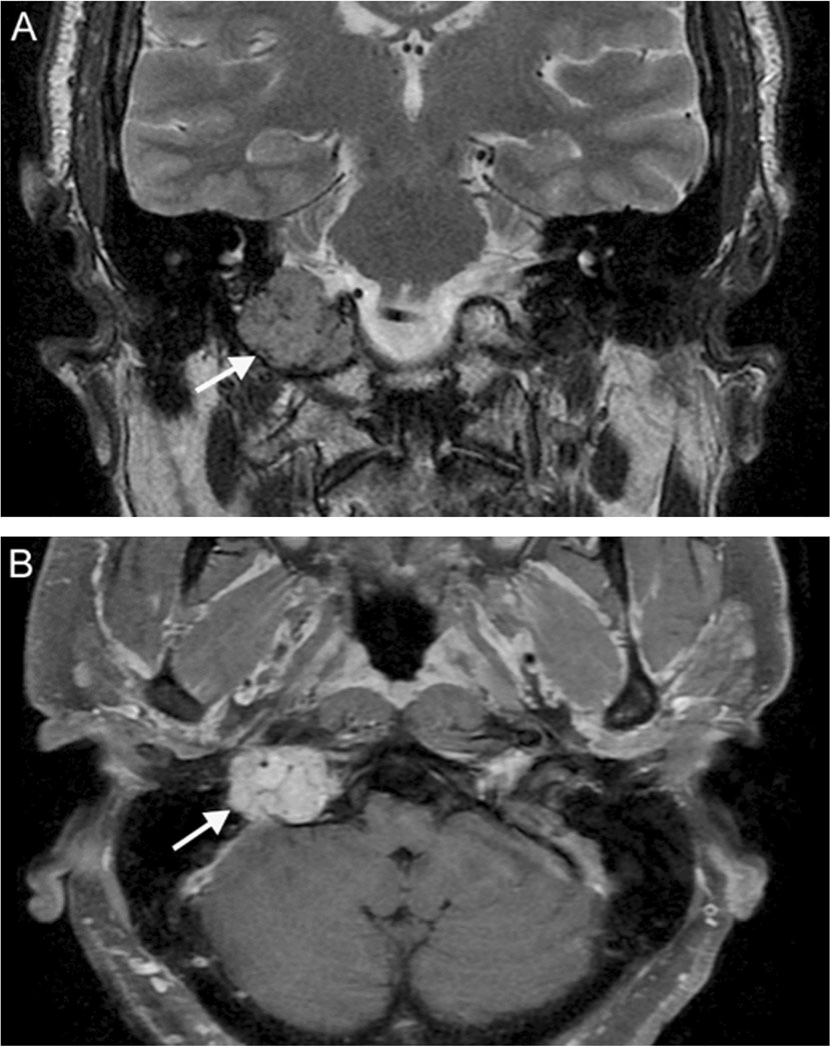
Jugular paraganglioma. A 55-year-old woman with relatively sudden onset hoarseness and dysphagia to liquids, who was found to have right vocal cord paralysis on laryngoscopy. Coronal T2 (**a**) and axial T1 post-contrast (**b**) MR images reveal a T2-isointense, avidly-enhancing lesion at the right jugular fossa (arrows) extending intracranially, with mild mass effect upon the right cerebellar hemisphere. Foci of low signal (“pepper”) represent flow voids, reflecting hypervascularity. Based on location and appearance, findings are consistent with a jugular paraganglioma (glomus jugulare tumor)

**Fig. 14:**
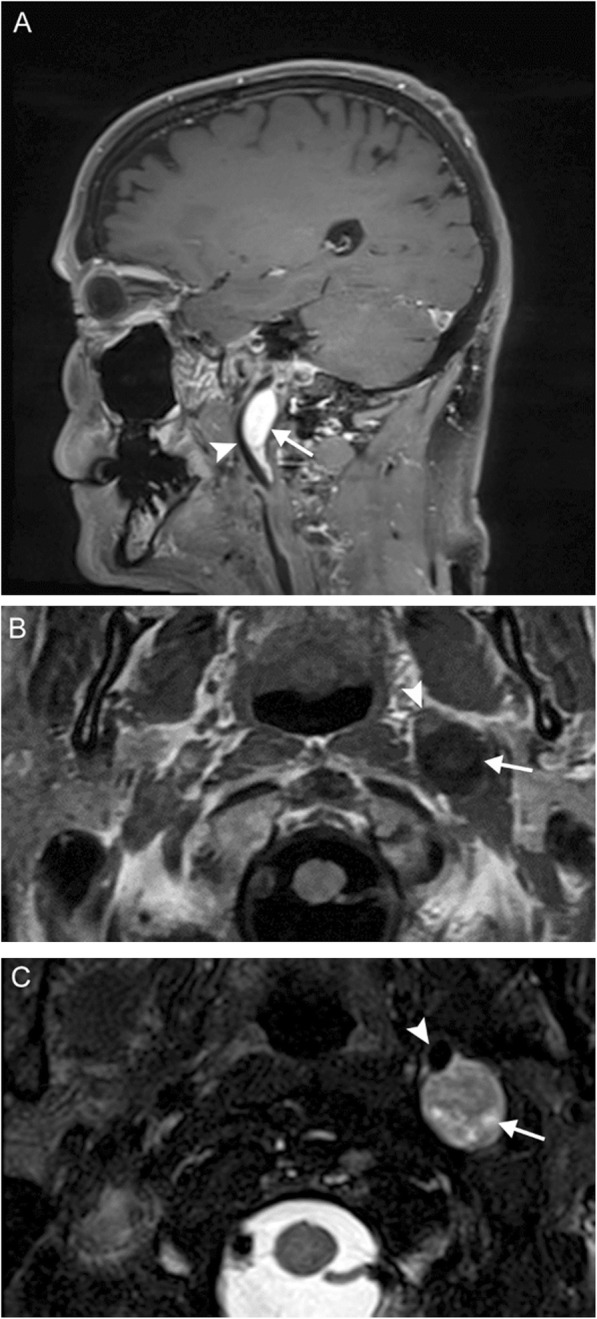
Vagal paraganglioma. A 67-year-old man presenting with raspy voice. Sagittal T1 post-contrast (**a**), axial T1 (**b**), and axial T2 (**c**) MR images reveal a T1-hypointense, avidly enhancing lesion with heterogeneous areas of high T2 signal (arrows) centered within the left carotid space. The internal carotid artery is displaced anteromedially (arrowheads). Pathology was consistent with a vagal paraganglioma (glomus vagale tumor)

**Fig. 15:**
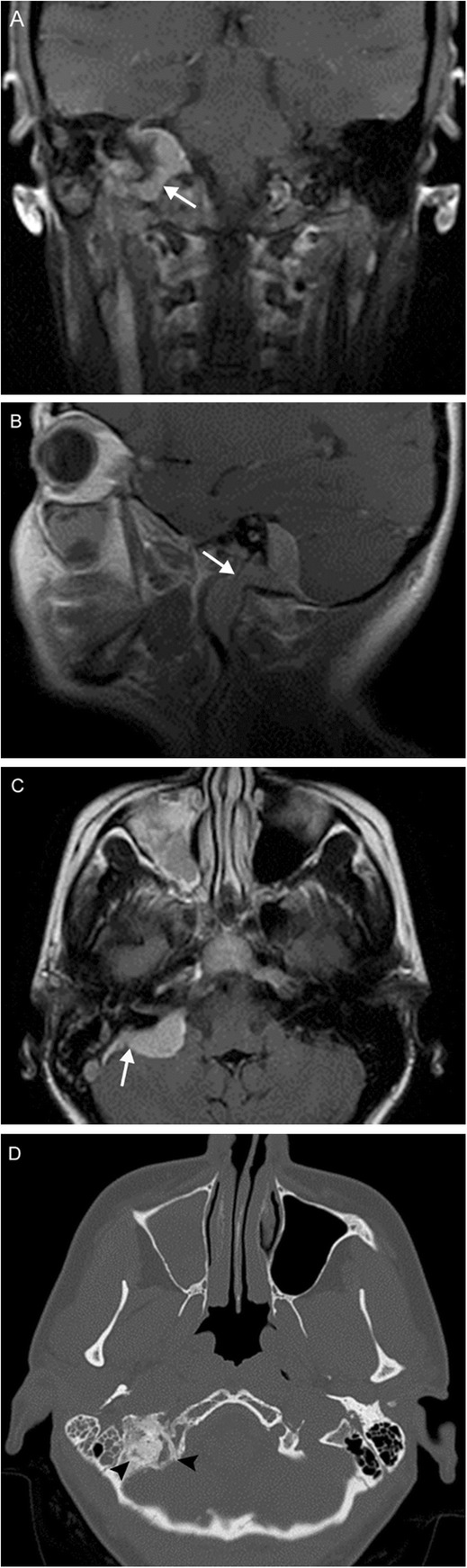
Meningioma. A 45-year-old woman initially presenting with right sensorineural loss, followed by headache, dysphagia, dysphonia, imbalance, and vasovagal syncope. Coronal (**a**), sagittal (**b**), and axial (**c**) post-contrast T1-weighted MR images reveal a dural-based mass at the right cerebellopontine angle (white arrows) extending into the internal auditory canal, as well as through the jugular foramen into the carotid space. Axial contrast-enhanced CT (**d**) reveals diffuse hyperostosis of the right temporal bone with opacification of the mastoid air cells (black arrowheads). Overall, findings are consistent with a meningioma. Incidental severe right maxillary sinus mucosal thickening is also seen

**Fig. 16:**
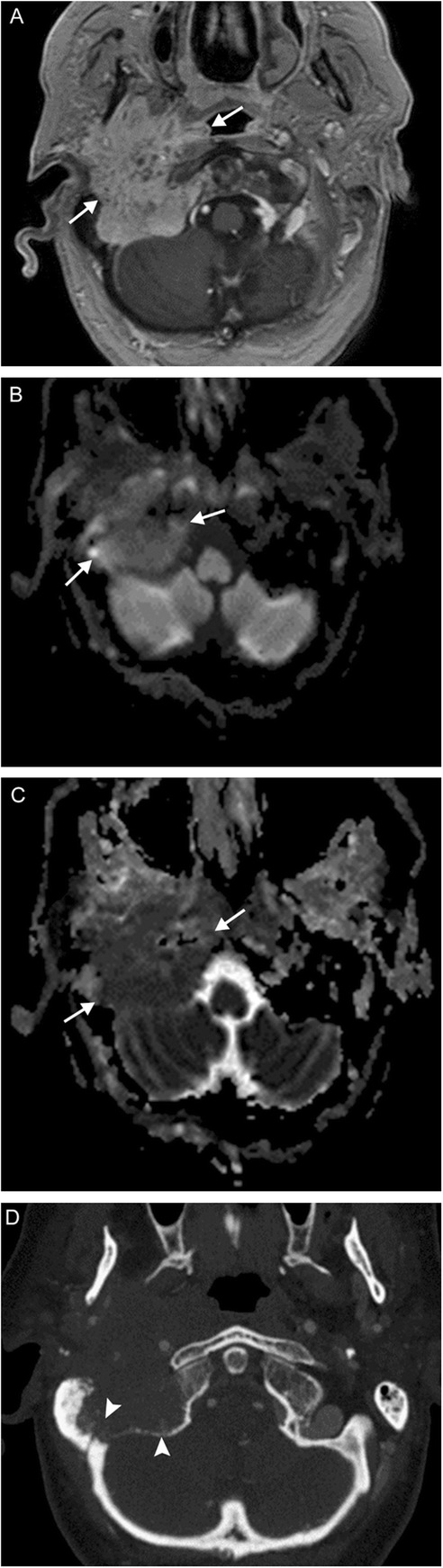
Skull base metastasis. A 63-year-old woman with several weeks of right-sided ear infection and hoarseness, followed by sudden onset right facial droop. Axial post-contrast T1-weighted (**a**) and DWI/ADC (**b**, **c**) MR images reveal a large enhancing infiltrative lesion with diffusion restriction at the skull base (arrows). Axial CTA image (**d**) demonstrates osseous destruction and obliteration of the right internal jugular vein (arrowheads). This was found to be metastatic adenocarcinoma of unknown primary

Multiple lower cranial neuropathies should raise the suspicion for a skull base tumor or other lesion, such as an occipital condylar fracture in the setting of trauma. The most common primary tumors are paraganglioma (Figs. 12, 13, and 14), schwannoma, and meningioma (Fig. 15), while the most common metastases (Fig. 16) are from lung, breast, and prostate carcinomas. Other malignancies that can involve the skull base include lymphoma and multiple myeloma.

### Paragangliomas

Paragangliomas, or glomus tumors, are slow-growing vascular neoplasms that arise from the neural crest, histologically equivalent to pheochromocytomas of the adrenal gland, and are intimately associated with neurovascular structures. Although extra-adrenal paragangliomas are most common in the head and neck, they are rare, accounting for approximately 0.5% of all head and neck tumors. Most are sporadic, but with new discoveries of genetic markers, it is now thought that up to 30% may be familial. Incidence slightly favors women, and diagnosis is typically during the third through fifth decades. Most are benign, and malignancy is determined by the presence of metastatic lesions as there is no histologic difference. The vast majority are not vasoactive; symptomatology is usually determined by location.

Paragangliomas are named for their location of origin; the four most common primary sites in the head and neck are at the tympanic membrane (glomus tympanicum), jugular fossa (glomus jugulare), within the carotid sheath (glomus vagale), and at the carotid bifurcation (glomus caroticum or carotid body tumor). These tumors have the propensity for trans-spatial spread; for example, a glomus jugulotympanicum tumor involves both the skull base and middle ear. Glomus jugulare tumors tend to present as pulsatile tinnitus and hearing loss, although lower cranial nerve symptoms such as hoarse voice can be present as well. Glomus vagale tumors occur along the vagus nerve and are accompanied by VCP approximately half of the time.

Paragangliomas can be imaged with a number of modalities, including CT, MRI, and nuclear medicine studies [18]. The intimate association of glomus tympanicum and jugulare with structures of the temporal bone and skull base lends well to the use of CT; aggressive tumors may result in bony destruction with a moth-eaten appearance. On MRI, these tumors are classically described as “lightbulb bright” on T2-weighted imaging due to extensive vascularity and “salt and pepper” due to flow voids. Avid enhancement is seen with both iodinated and gadolinium contrast. Due to their avidity for tumors of neuroendocrine origin, a number of radionuclides can be used to identify paragangliomas: ^111^In-pentreotide and 68Ga-DOTATATE for somatostatin receptor positivity, ^18^F-fluorodeoxyglucose (^18^F-FDG) for metabolic activity, and ^123^I-metaiodobenzylguanidine (^123^I-MIBG) and ^18^F-fluorodopamine (^18^F-FDA) for catecholamine production.

## Lesions of the carotid space

The carotid space, also known as the poststyloid compartment of the parapharyngeal space, is one of the deep spaces of the neck, spanning from the skull base to the aortic arch. It is enclosed by all three layers of the deep cervical fascia, termed the carotid sheath. It is bordered anterolaterally by the sternocleidomastoid muscle, anteromedially by the parapharyngeal space and visceral space, and posteriorly by the prevertebral space. Its primary contents are the carotid artery, internal jugular vein, and vagus nerve, but also contains other lower cranial nerves (CN IX, XI, XII) superiorly, sympathetic nerves (cervical sympathetic plexus anteriorly and ansa cervicalis posteriorly), and the deep cervical lymph node chain. Lesions tend to be neurovascular in origin but can arise from any of these components [19]; given the confined space, these can all affect the vagus nerve and lead to dysphonia.

### Benign nerve sheath tumors

**Fig. 17:**
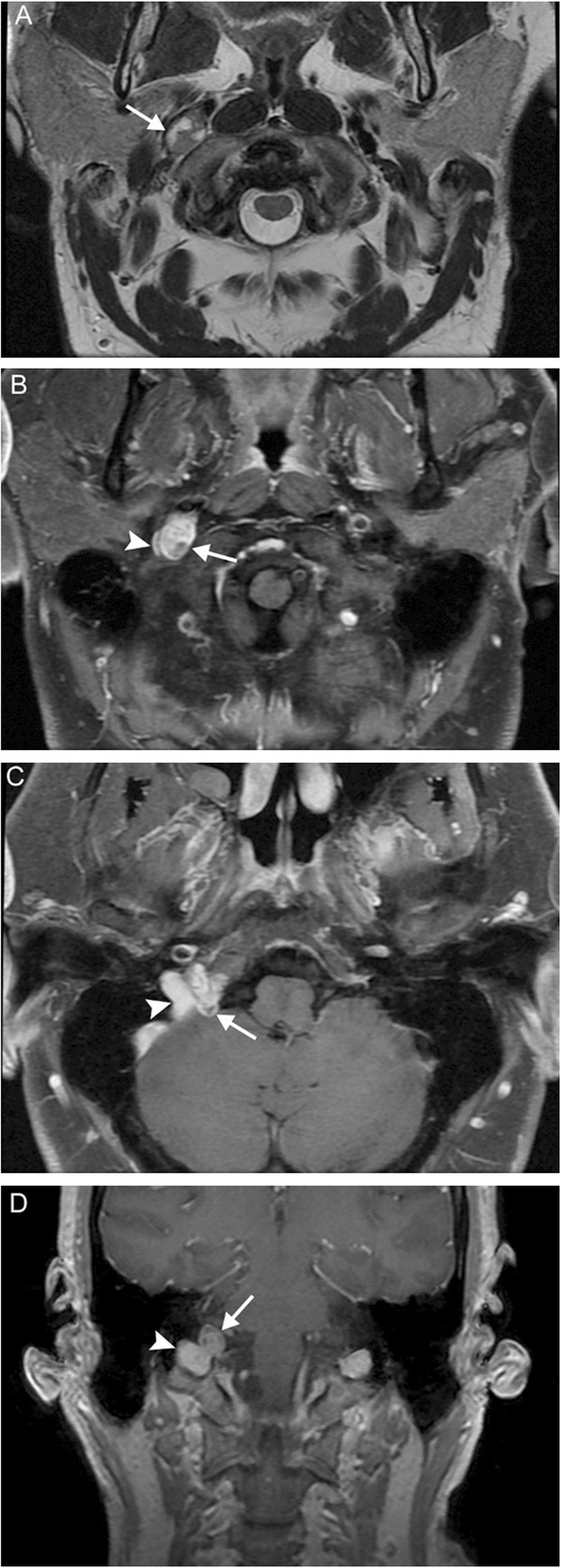
Vagal Schwannoma. A 53-year-old man with months of cough and hoarseness, thought to be reflux but not improved with proton pump inhibitor. Axial T2 (**a**), axial post-contrast T1 (**b**, **c**), and coronal post-contrast T1 (**d**) MR images reveal a well-circumscribed enhancing mass (arrows) with cystic components extending through the right jugular foramen (note the 'waist') into the carotid space. The right internal jugular vein (arrowheads) is compressed within the carotid sheath. Findings are characteristic for a vagal schwannoma, with intracranial and extracranial extent

Schwann cells are the principle glial cells of the peripheral nervous system, myelinating peripheral nerves, while perineural fibroblasts comprise the perineurium, which is the connective tissue surrounding nerve fascicles. From these nerve sheath elements arise schwannomas and neurofibromas; these benign tumors can affect any peripheral nerve, including the cranial nerves [20, 21]. Benign nerve sheath tumors of the head and neck are mostly schwannomas, although neurofibromas are seen in syndromic cases such as neurofibromatosis type I. These tumors can be difficult to distinguish from each other based on clinical or imaging examination alone—both will present as a well-circumscribed encapsulated mass with avid (usually homogenous) enhancement (Fig. 17)—so these entities are considered together when forming a differential diagnosis. They can be differentiated from paragangliomas by their intimate association with the peripheral nerve and lack of flow voids.

Of the cranial nerve schwannomas, the vast majority (90%) are of the vestibular branch of the vestibulocochlear nerve (CN VIII), but can also affect the trigeminal nerve (CN V), facial nerve (CN VII), or lower cranial nerves (CN IX-XII). Vagal nerve schwannomas have a slight female predominance and usually present in middle age, although they can occur at any age. These tumors most often present as an asymptomatic slow-growing palpable neck mass; however, the second most common presentation is hoarseness. The most specific sign for vagal nerve schwannoma is paroxysmal cough induced by palpation or manipulation of the mass, due to stimulation of the vagal nerve [22]. Treatment is surgical resection; preservation of the vagus nerve can be difficult; however, schwannomas tend to be more easily resected since they arise from the myelin surrounding the entire nerve and are often eccentric to the nerve itself, rather than neurofibromas which arise from the perineurium surrounding individual fascicles and thus can be entwined in the fibers [20, 22].

## Thyroid disease

**Fig. 18:**
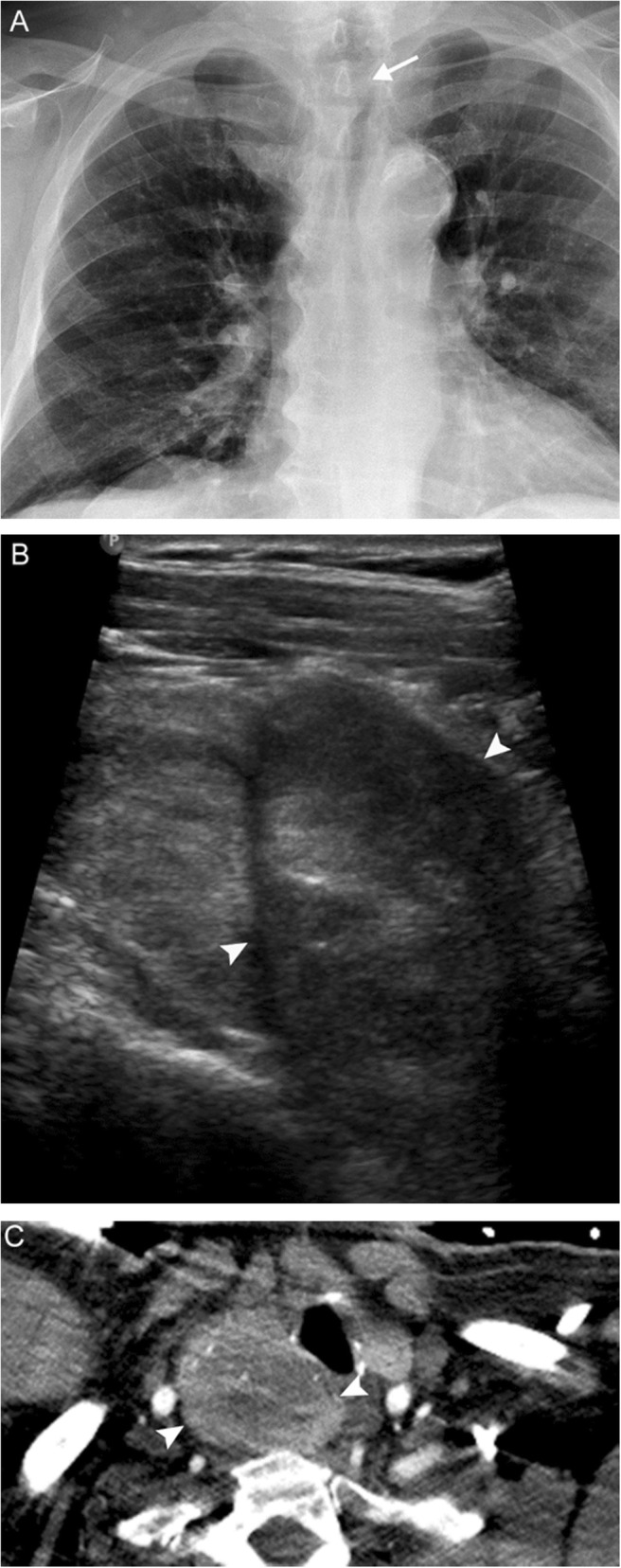
Thyroid carcinoma. A 78-year-old man presenting with persistent cough, hoarse voice, and dyspnea. Chest radiograph (**a**) reveals a right paratracheal mass indenting the subglottic trachea (white arrow). Subsequent neck US (**b**) and chest CTA (**c**) reveal a solid heterogenous mass with calcifications (white arrowheads), which narrows but does not invade the glottic/subglottic airway. Fine needle aspiration revealed follicular thyroid carcinoma

The thyroid gland resides in the anterior infrahyoid neck below the larynx and anterior to the thyroid cartilage. The whole spectrum of thyroid disease may manifest with dysphonia due to both location and endocrine function. The gland itself may become enlarged in such conditions as multinodular goiter, thyroiditis, or carcinoma (Fig. 18), stretching the recurrent laryngeal nerve which courses behind the gland. Thyroiditis can result in a sore throat and raspy voice similar to pharyngitis. Severe hyperthyroidism may have tremulous voice. Severe hypothyroidism with myxedema can affect any part of the body, including the vocal cords (a form of Reinke’s edema). Treatment of underlying thyroid disease may or may not reverse any dysphonia.

## Lesions of the mediastinum

**Fig. 19:**
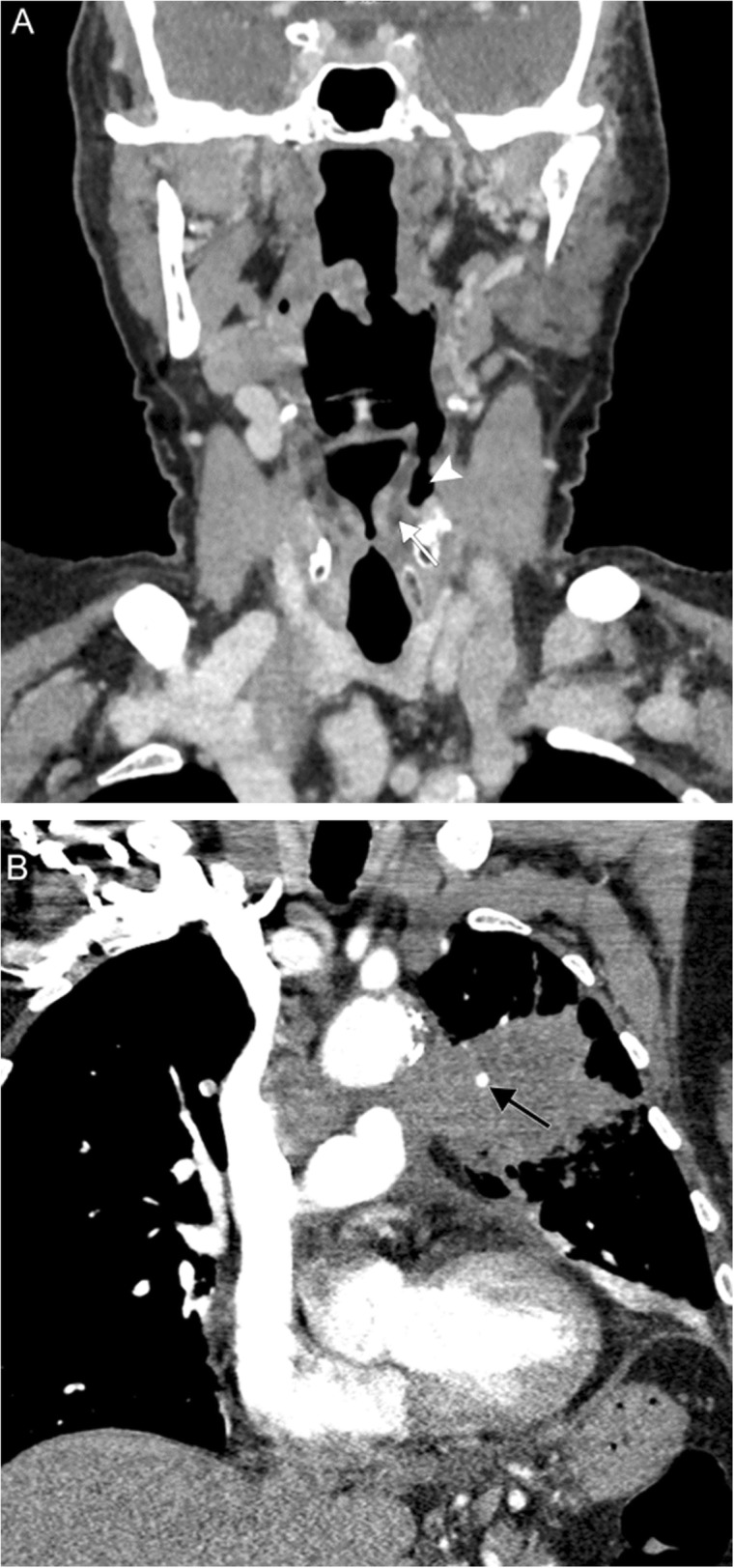
Lung cancer. A 79-year-old woman with 55-pack-year smoking history presenting with hoarse voice and chronic vomiting. Coronal contrast-enhanced images of the larynx (**a**) and lungs/mediastinum (**b**) reveal medialization of the left vocal fold (white arrow) with dilation of the ipsilateral piriform sinus (white arrowhead). A large mass centered in the left upper lobe extends into the aortopulmonary window and also encases a vessel (black arrow). These findings are consistent with left vocal cord paralysis due to compression of the left recurrent laryngeal nerve in the setting of stage IV lung cancer

**Fig. 20:**
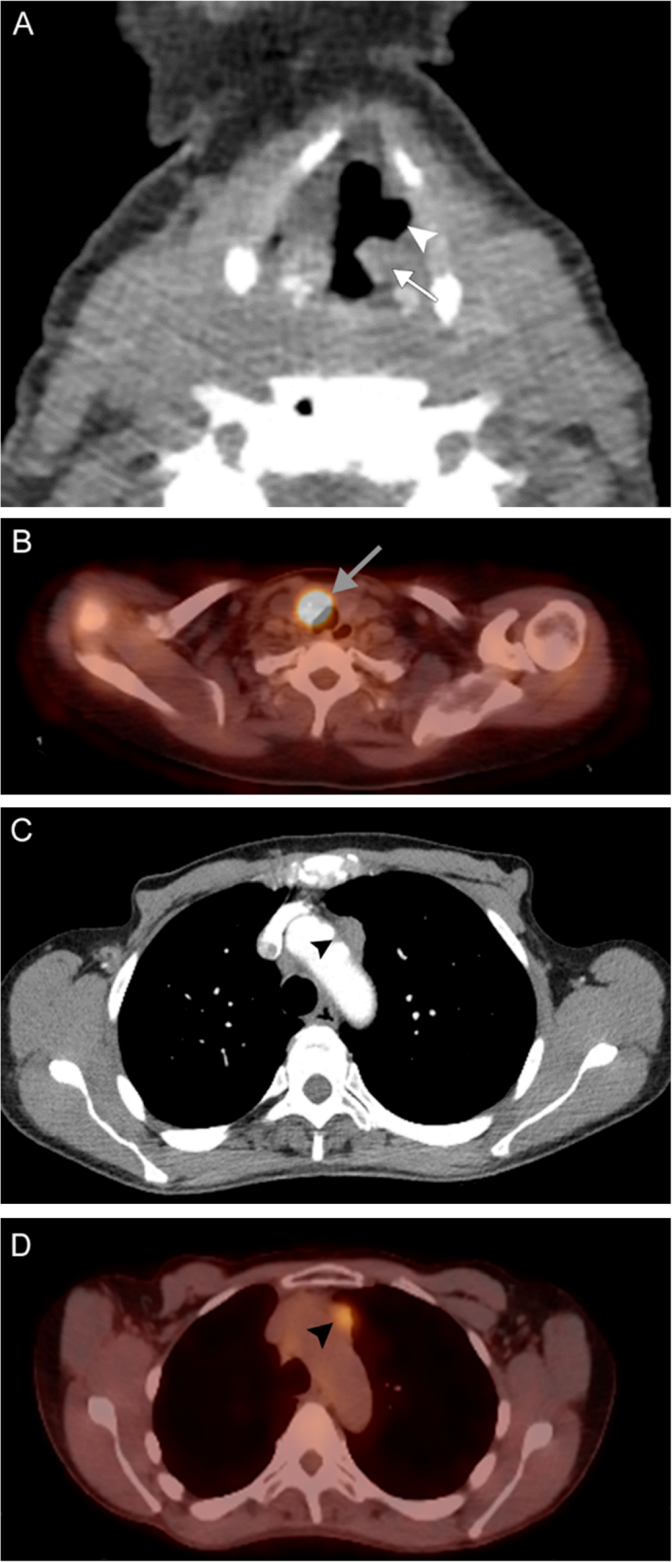
Metastatic mediastinal lymphadenopathy. A 59-year-old woman with right breast cancer status post chemoradiation completed 5 years prior presents with rapidly progressive hoarseness. An axial non-contrast CT image (**a**) demonstrates anteromedial rotation of the left posterior vocal fold and arytenoid cartilage (white arrow) with associated left-sided dilatation of the laryngeal ventricle (white arrowhead). Associated PET image (**b**) demonstrates compensatory FDG uptake in the contralateral vocal fold (gray arrow). Contrast-enhanced CT (**c**) and PET (**d**) images more inferiorly reveal hypermetabolic prevascular lymphadenopathy in the superior mediastinum (black arrowheads). Overall, these findings are consistent with left vocal cord paralysis due to compression of the left recurrent laryngeal nerve by a metastatic prevascular lymph node

**Fig. 21:**
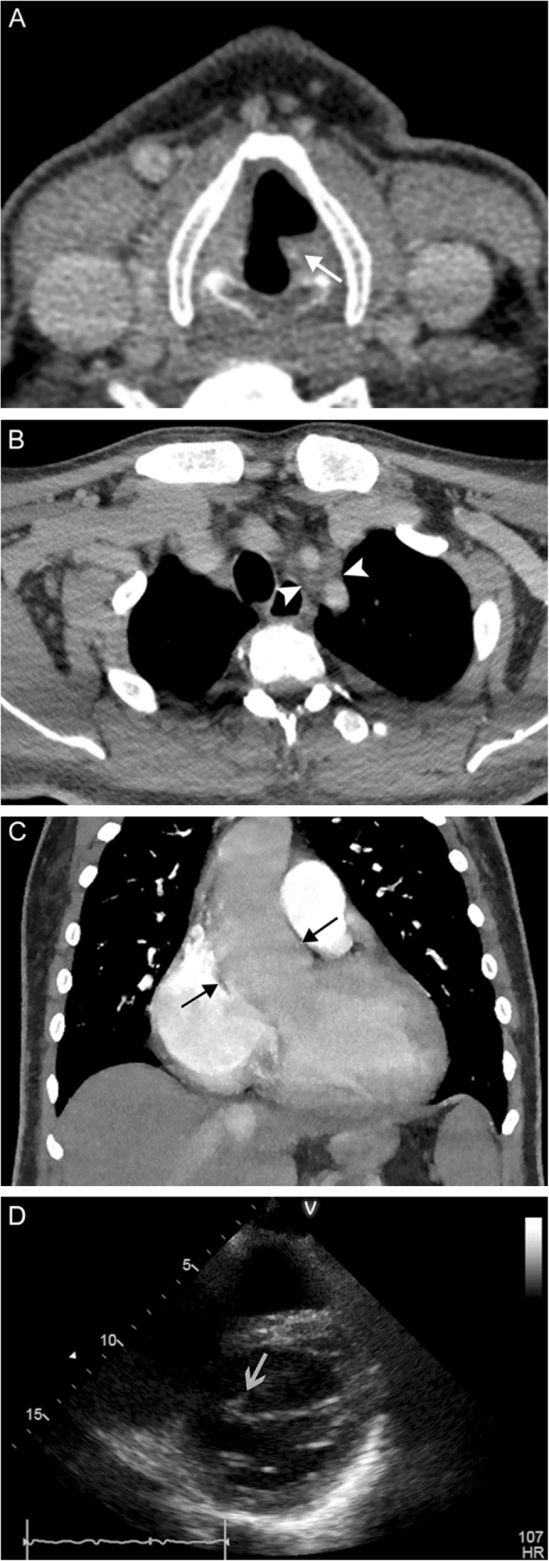
Endocarditis. A 41-year-old previously healthy man presents with intermittent hoarse voice and unexpected weight loss. Axial contrast-enhanced CT images through the larynx (**a**) and mediastinum (**b**) reveal left vocal fold rotation (white arrow) and lymphadenopathy surrounding the left carotid and subclavian arteries (white arrowheads). A coronal CTA image (**c**) reveals an ascending aortic aneurysm (black arrows). Echocardiogram (**d**) reveals a bicuspid valve with vegetations (gray arrow) and severe aortic stenosis. Overall, findings are consistent with compression/stretching of the left recurrent laryngeal nerve due to reactive lymphadenopathy and a dilated aortic arch in the setting of endocarditis

**Fig. 22:**
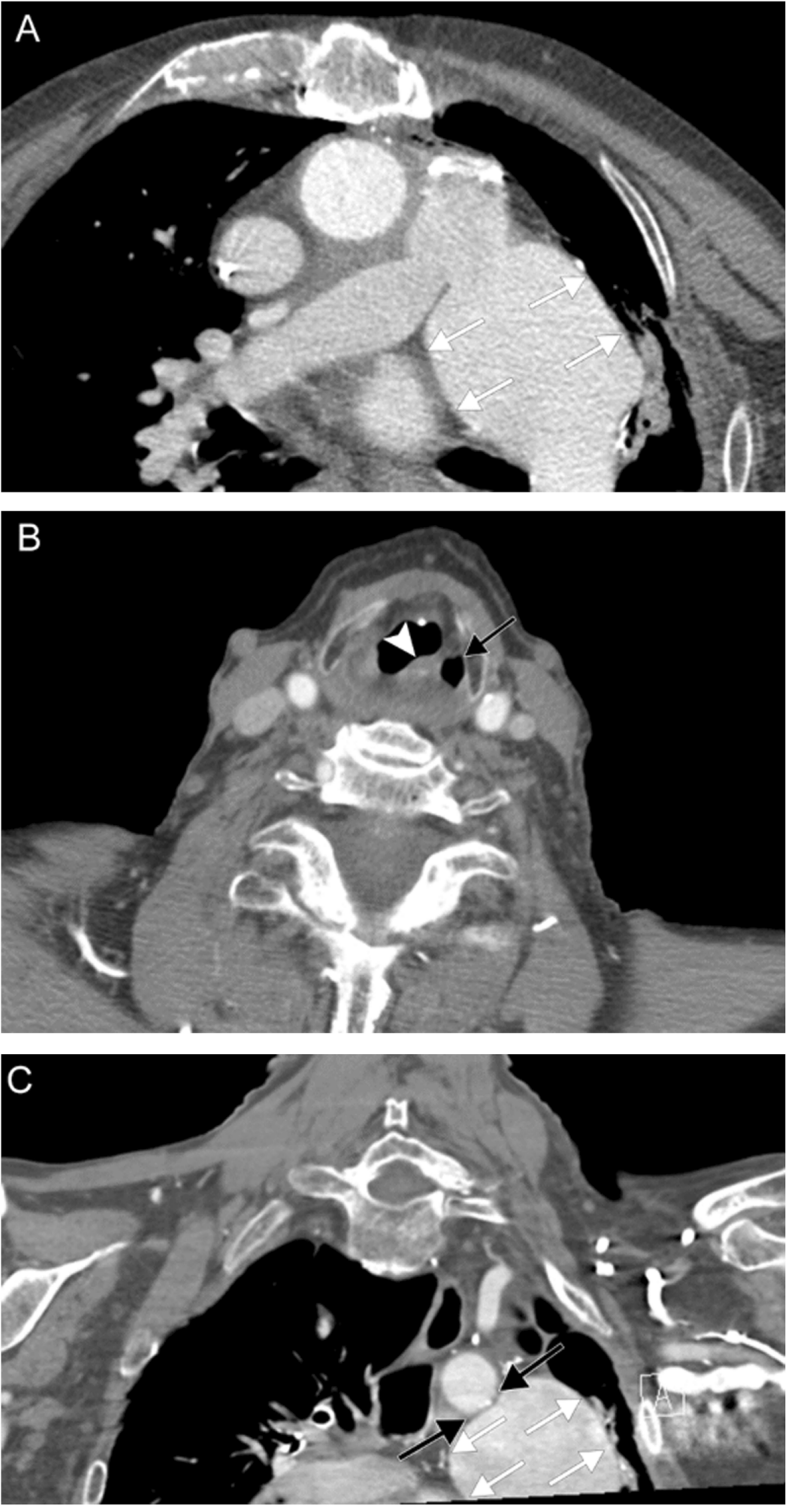
Pulmonary artery aneurysm. A 78-year-old man with advanced congestive heart failure secondary to congenital pulmonary artery stenosis status post multiple corrective surgeries, now presenting with progressive shortness of breath and speaking difficulty despite adequate management of heart failure. Axial CTA image (**a**) through the mediastinum reveals aneurysmal dilatation of the left pulmonary artery (white arrows). An axial CTA image through the larynx (**b**) reveals thickening of the left aryepiglottic fold (white arrowhead) and dilatation of the left piriform sinus (black arrow). A coronal CTA image (**c**) demonstrates effacement of the aortopulmonary window (black arrows) by the pulmonary artery aneurysm (white arrows) with presumed compression of the left recurrent laryngeal nerve

The RLNs course within the upper mediastinum, placing them at risk for injury due to a number of mediastinal processes. Numerous pathologies of the cardiovascular structures of the mediastinum (pulmonary artery aneurysm) can present with VCP as can any number of infectious/inflammatory and neoplastic etiologies with mediastinal lymphadenopathy (endocarditis) (Figs. 19, 20, 21, and 22). Mediastinal lesions typically cause VCP as a result of mass effect.

## Traumatic/iatrogenic nerve injury

**Fig. 23:**
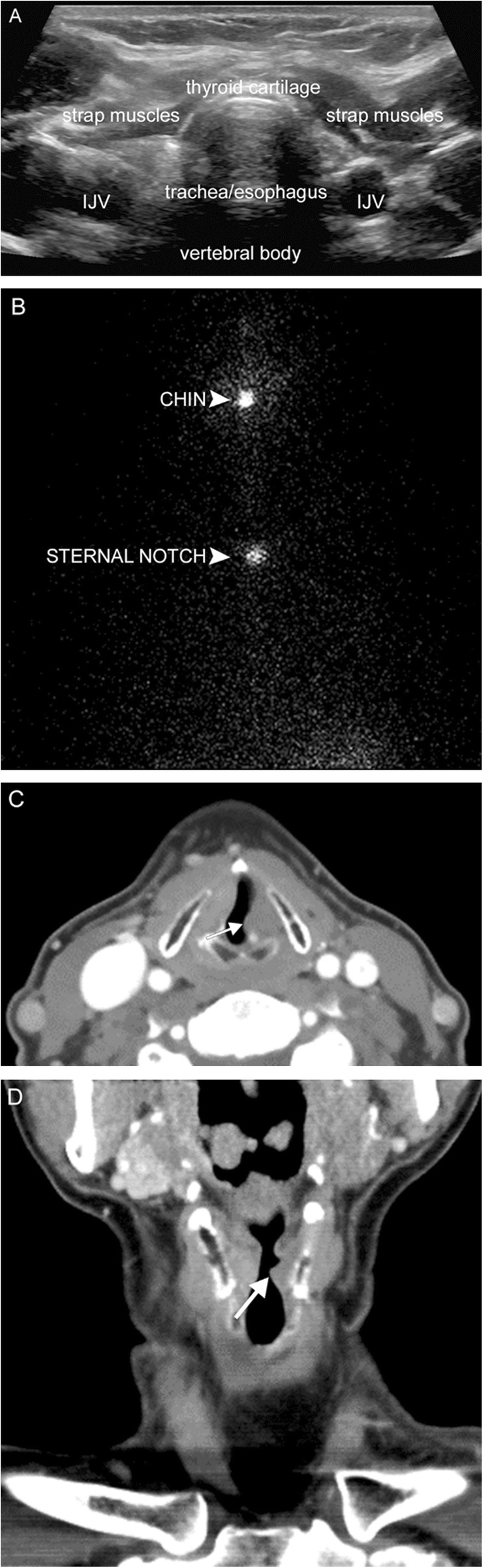
Thyroidectomy. A 63-year-old woman status post total thyroidectomy and radioablation for papillary thyroid carcinoma. Neck ultrasound (**a**), I-131 whole body scintigraphy (**b**), and axial/coronal post-contrast CT images (**c**, **d**) demonstrate no evidence for residual thyroid tissue in the thyroid bed. In addition, the left vocal fold is medialized (arrow) with dilatation of the left laryngeal ventricle, suggesting vocal cord paralysis due to iatrogenic injury

**Fig. 24:**
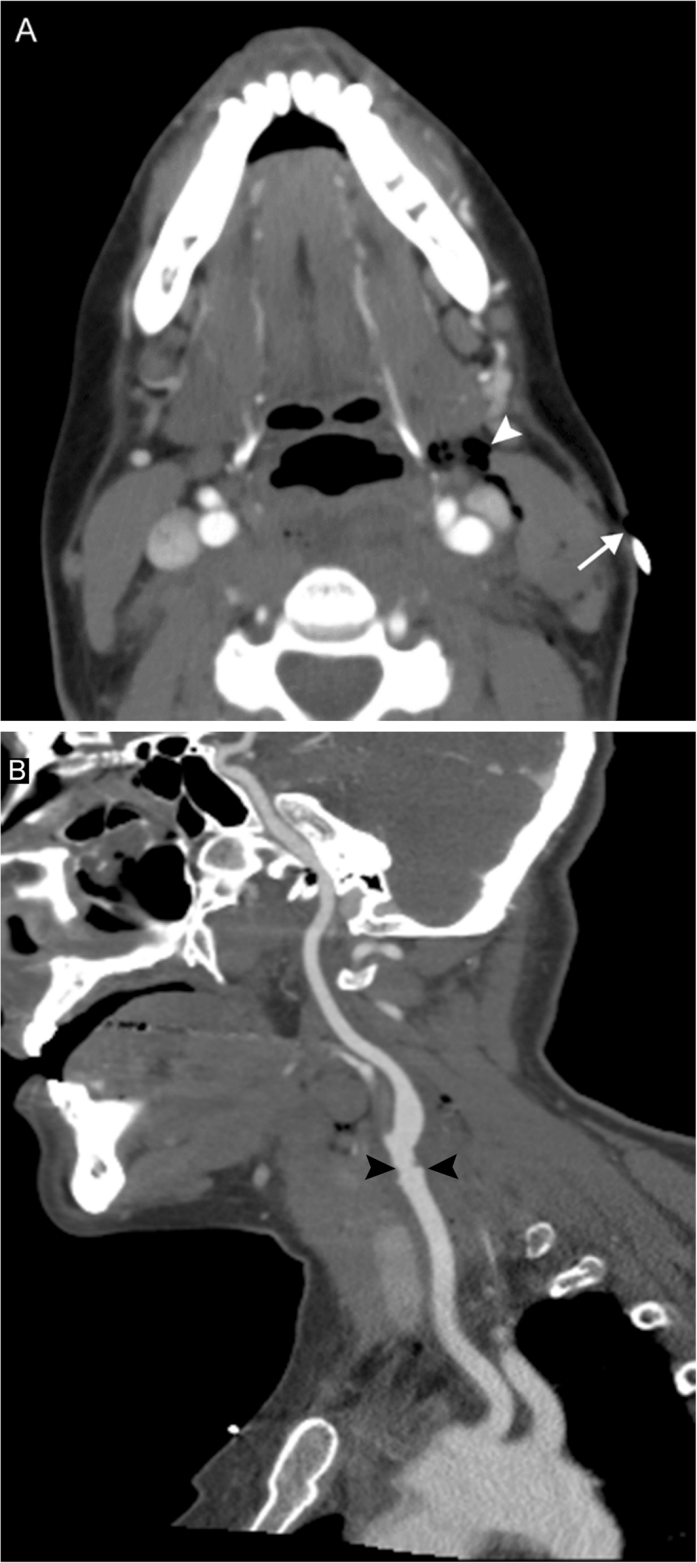
Carotid space trauma. A 33-year-old woman who sustained a stab wound to the neck, subsequently noted to have a weak voice; laryngoscopy confirmed vocal cord paralysis. Axial CTA of the neck (**a**) reveals a radiopaque marker at the entry site (white arrow); air is seen within the deep soft tissues anterior to the left carotid sheath contents (white arrowhead), indicating violation of the left carotid space. 3D-volume-rendered CTA image (**b**) of the left carotid artery shows irregular caliber and focal outpouching of the distal left common carotid artery (black arrowheads), representing pseudoaneurysm. Findings are consistent with left vocal cord paralysis due to vagal nerve injury

The vagus and recurrent laryngeal nerves are susceptible to iatrogenic injury due to their long course through the neck and mediastinum and proximity to several structures. Iatrogenic VCP brings to mind traumatic intubation due to subluxation or dislocation of the cricoarytenoid joint [23]; however, the most common cause of iatrogenic VCP is actually injury during surgeries such as thyroidectomy (Fig. 23), carotid endarterectomy, and anterior cervical spinal surgery. Although rare, dysphonia can be seen secondary to blunt or penetrating injury [24–27] either from direct injury to the larynx, or injury to CN X—which is vulnerable as it courses through the neck given its mobility and lack of surrounding protective structures (Fig. 24).

## Summary

Dysphonia can result from a wide range of entities occurring in the larynx as well as a number of different anatomic sites ranging from the brainstem to the mediastinum:

- Laryngeal carcinoma often has a better prognosis than a similarly staged head and neck squamous cell carcinoma of another primary site. Presenting symptoms depend on the laryngeal subsite involved.
- Infiltrative laryngeal lesions such as papillomatosis and amyloidosis are benign but progressive, resulting in dysphonia and/or airway obstruction and frequently requiring multiple treatments due to extent and recurrence.
- A well-positioned brainstem lesion may affect either the inferior olivary nucleus (hypertrophic olivary degeneration) or nucleus ambiguous (lateral medullary syndrome), leading to classic constellations of higher-order neuronal symptoms including dysphonia.
- Multiple lower cranial neuropathies should trigger search for a lesion at the skull base, particularly at the jugular foramen.
- The vagus nerve is confined within the carotid sheath as it courses down the neck, rendering it susceptible to injury from neck trauma or neck mass.
- The whole spectrum of thyroid disease can result in dysphonia due to the gland’s position below the larynx.
- The recurrent laryngeal nerve can be either stretched or compressed by lesions in the superior mediastinum, resulting in vocal cord paralysis.

Knowledge of these different lesions put into clinical context will help the radiologist narrow the differential diagnosis and guide the clinician to proper ordering of tests.

## Abbreviation

CN: Cranial nerve
CT: Computed tomography
CTA: Computed tomography angiography
FDG: Fluorodeoxyglucose
HOD: Hypertrophic olivary degeneration
HPV: Human papilloma virus
ION: Inferior olivary nucleus
MIBG: Metaiodobenzylguanidine
MR: Magnetic resonance
MRA: Magnetic resonance angiography
PET: Positron emission tomography
PICA: Posterior inferior cerebellar artery
RLN: Recurrent laryngeal nerve
SCC: Squamous cell carcinoma
TOF: Time of flight
VCP: Vocal cord paralysis
